# Investigating and Learning Lessons from Early Experiences of Implementing ePrescribing Systems into NHS Hospitals: A Questionnaire Study

**DOI:** 10.1371/journal.pone.0053369

**Published:** 2013-01-15

**Authors:** Kathrin Cresswell, Jamie Coleman, Ann Slee, Robin Williams, Aziz Sheikh, ePrescribing Programme Team

**Affiliations:** 1 The School of Health in Social Science, The University of Edinburgh, Edinburgh, United Kingdom; 2 School of Clinical and Experimental Medicine, The University of Birmingham, Birmingham, United Kingdom; 3 Centre for Population Health Sciences, The University of Edinburgh, Edinburgh, United Kingdom; 4 Institute for the Study of Science, Technology and Innovation, The University of Edinburgh, Edinburgh, United Kingdom; University of New South Wales, Australia

## Abstract

**Background:**

ePrescribing systems have significant potential to improve the safety and efficiency of healthcare, but they need to be carefully selected and implemented to maximise benefits. Implementations in English hospitals are in the early stages and there is a lack of standards guiding the procurement, functional specifications, and expected benefits. We sought to provide an updated overview of the current picture in relation to implementation of ePrescribing systems, explore existing strategies, and identify early lessons learned.

**Methods:**

A descriptive questionnaire-based study, which included closed and free text questions and involved both quantitative and qualitative analysis of the data generated.

**Results:**

We obtained responses from 85 of 108 NHS staff (78.7% response rate). At least 6% (n = 10) of the 168 English NHS Trusts have already implemented ePrescribing systems, 2% (n = 4) have no plans of implementing, and 34% (n = 55) are planning to implement with intended rapid implementation timelines driven by high expectations surrounding improved safety and efficiency of care. The majority are unclear as to which system to choose, but integration with existing systems and sophisticated decision support functionality are important decisive factors. Participants highlighted the need for increased guidance in relation to implementation strategy, system choice and standards, as well as the need for top-level management support to adequately resource the project. Although some early benefits were reported by hospitals that had already implemented, the hoped for benefits relating to improved efficiency and cost-savings remain elusive due to a lack of system maturity.

**Conclusions:**

Whilst few have begun implementation, there is considerable interest in ePrescribing systems with ambitious timelines amongst those hospitals that are planning implementations. In order to ensure maximum chances of realising benefits, there is a need for increased guidance in relation to implementation strategy, system choice and standards, as well as increased financial resources to fund local activities.

## Introduction

A number of international benchmark studies have demonstrated that prescribing errors are common and are responsible for considerable – potentially avoidable – morbidity and mortality [1]–[3]. Given the increasing complexity of prescribing decisions, the risk of prescribing-related iatrogenic harm is likely to increase yet further. Improving the quality and safety of prescribing, as well as optimising the use of medicines throughout the health sector is therefore now firmly established as a priority area throughout much of the economically-developed world, including the United Kingdom (UK). Electronic prescribing (or ePrescribing) is seen as one way to help deliver on this priority issue.

ePrescribing systems involve *“the utilisation of electronic systems to facilitate and enhance the communication of a prescription or medicine order, aiding the choice, administration and supply of a medicine through knowledge and decision support and providing a robust audit trail for the entire medicines use process*” [4]. As such, although systems vary in sophistication, they can amongst other things help to prevent duplicate prescribing, minimise the risk of contraindicated prescribing (e.g. due to drug-drug interactions), reduce dosing errors, support more cost effective prescribing, and facilitate adherence to formulary-based recommendations [5]–[11].

However, realising these benefits is far from straightforward. This is particularly true for UK hospitals, where ePrescribing systems are to date not routinely used. These technologies can initially be extremely disruptive, changing established ways of working and patients’ experiences of care [12], [13]. They can furthermore result in unintended consequences, which may include new kinds of error and thus potential threats to patient safety. Koppel and colleagues, for example, reported how fragmented basic ePrescribing system displays prevented a coherent view of patients’ medication and how the separation of functions facilitated double dosing [14]. Similarly, it has been found that many systems can produce clinically spurious alerts [15], [16]. This frustrates end-users and result in alerts commonly being over-ridden or ignored. More recent work has highlighted potentially serious treatment delays that can inadvertently be associated with the introduction of ‘hard stops’ to reduce risk of serious prescribing errors [17], [18]. It is therefore important that such systems are carefully selected and implemented to maximise the likelihood of benefit to patients. However, existing technologies vary considerably in functionality, level of inter-operability and costs. The variety of systems and stakeholders involved complicates organisational decision making regarding the choice of system and implementation strategies. This is further compounded by a lack of standards guiding the procurement, functional specifications, expected benefits and strategies for implementation of these systems [19], [20].

The UK context is unusual as there were previous attempts to introduce national health information systems through the National Programme for Information Technology (NPfIT), characterised by a centrally-led procurement and implementation model of selected software systems (that were subsidised by central resources) [21], [22]. The strategic direction has, however, recently changed to allow increased input of local organisations in decision making, which means that healthcare organisations are now faced with a wider range of choices and may lack central direction relating to procurement and implementation of systems [21], [22].

Although ePrescribing systems are very well established in UK primary care, the transfer of electronic data between hospitals and primary care settings is to date limited [15], [19], [23], [24]. The major primary care systems in the UK are EMIS, Vision, TPP SystmOne and GPASS [23], [24], the ePrescribing functionality being an integral feature of the electronic health record system.

To inform our on-going national evaluation of the introduction of ePrescribing systems into English National Health Service (NHS) hospitals, and building on previous work that reported considerable interest in investing in ePrescribing systems [20], [25], we undertook a scoping study in which we sought to: 1) provide an updated overview of the current picture in relation to implementation of ePrescribing systems; 2) explore existing implementation strategies; and 3) identify early lessons learned.

## Materials and Methods

### Ethical Considerations

This work is part of a national service evaluation investigating the implementation and adoption of ePrescribing systems in English hospitals [25]. Participants gave verbal informed consent to participate.

### Design and Questionnaire Development

We undertook a questionnaire-based descriptive study with a combination of closed and free text questions, which involved both quantitative and qualitative analysis of the data generated. The design of our questionnaire was informed by the existing literature surrounding information technology (IT) implementations in healthcare settings [8], [13], [26]–[29], our own experiences from implementing and evaluating related applications [20], [25], and a questionnaire from a scoping study of planned implementations conducted in 2010 [20]. We devised a range of structured and semi-structured questions enquiring about participant demographics, system functionality and anticipated implementation timelines. In addition, we included a range of open-ended questions relating to implementation strategies, anticipated changes, and lessons learned to date. A full version of the questionnaire can be viewed in Figure S1. Iterative development and piloting with clinical and academic colleagues, allowed refinement of the included items over a period of two months. This resulted in changes to the ordering of items and phrasing of some questions.

### Distribution of the Questionnaire

As with our previous study, questionnaires were included in the delegate packs of attendees at a national conference on the implementation of ePrescribing systems into English NHS hospitals hosted by the research team in March 2012. The conference aimed to provide a national platform for networking of interested parties and discussion of experiences to date [25]. Delegates included 150 representatives from a range of NHS Trusts, system suppliers, and academic institutions. All attending NHS staff (n = 108) were encouraged to complete the questionnaire in a designated slot during the opening session of the conference. Completed forms were collected by the research team before delegates left this session.

### Data Handling and Analysis

Questionnaires were numbered consecutively and numerical, categorical and qualitative data were entered in a Microsoft Access database. Categorical and numerical data relating to participant demographics, system characteristics and capabilities (Section 1 and the first four items of Section 2, see Figure S1) were then exported into Microsoft Excel to calculate descriptive statistics. Qualitative data surrounding implementation strategies, anticipated changes and lessons learned (the remainder of items in Section 2 and Section 3, see Figure S1) were exported into NVivo9 software [30]. Qualitative data were initially coded against headings of the questionnaire, followed by cross-sectional coding to extract cross-cutting themes [31]. This resulted in four overarching themes with corresponding sub-themes. These were chosen based on frequency of occurrence (i.e. mentioned by a large number of participants), and on the basis of significance (i.e. helping to explore underlying tensions).

## Results

The following paragraphs will explore both the quantitative and qualitative results in more detail. For these purposes, we have divided the results into two sections. We first report on quantitative data including participant demographics, numbers of planned/on-going implementations, and specified systems functionality. We then explore more cross-cutting themes extracted in the qualitative analysis relating to implementation strategies and early lessons learned.

**Figure 1:**
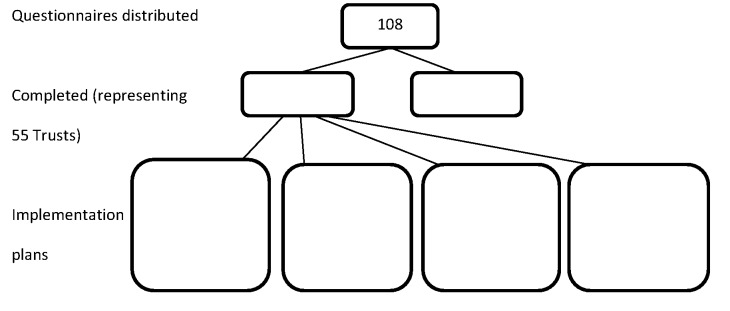
Flowchart illustrating numbers of participants, implementation status and systems.

We obtained responses from 85/108 NHS staff resulting in a response rate of 79% (Figure 1). Respondents came from a total of 55 different English NHS Trusts (out of a total of 168 throughout England, a Trust is a public sector organisation providing local services, there are 168 Trusts in the English NHS) and these comprised of: 47 Acute (hospital) Trusts (representing 85% of the total of Acute Trusts); seven Mental Health Trusts (representing 13%), and one Community Trust (representing 2%). Responses from the same Trust were combined to avoid double-counting.

The majority of respondents (n = 53, 62%) were pharmacists. They tended to be in senior positions such as Heads of Medicines Management, Chief Pharmacists, and ePrescribing Leads. The remainder included Managers (n = 22, 26%), Consultant (medical) Clinical Leads (n = 6, 7%), and Nurse Leads (n = 4, 5%).

### Planned/on-going Implementations, and Systems Functionality

#### Implementation of ePrescribing systems

Out of the 55 Trusts, 30 (55%) were planning to implement or in the process of procuring a system, 11 (20%) were currently implementing, 10 (18%) had already implemented, and four (7%) had no current plans of implementing an ePrescribing system (Table 1 and Figure 1).

**Table 1:** Implemented and plans to implement ePrescribing systems and system types in 55 NHS Trusts.

Already implemented(10 different Trusts)	Currently implementing(11 different Trusts)	Planning to implement/procuring(34 different Trusts)
4 JAC	2 Cerner	27 don’t know
1 Cerner	2 iCM	1 System C - CIS Chemocare
1 iCM	1 Galileo	1 Soarian Siemans Health
1 LastWord	1 JAC	1 Cerner
1 Mosaiq (Oncology)	1 MedChart	1 NWIS (built in-house)
1 PICS (built in-house)	1 System C	1 Ascribe
1 RiO	1 RiO	1 Ascribe or JAC
	1 TPP	1 Ascribe or RiO
	1 don’t know	

Of those that were currently implementing or had started procurement, the majority had or were expecting to initiate the process between 2008 and 2014 (median: 2012) and expected to finish between 2012 and 2019 (median: 2014). Trusts that had already implemented began between 1999 and 2009 (median: 2009) and finished between 2007 and 2012 (median: 2011).

The range of different systems implemented or planned to be implemented was relatively large and is summarised in Table 1. JAC had the largest number of completed implementations in four out of 10 Trusts (40%). Cerner was in the process of being implemented in two out of 11 Trusts (18%).

#### System functionality

**Figure 2:**
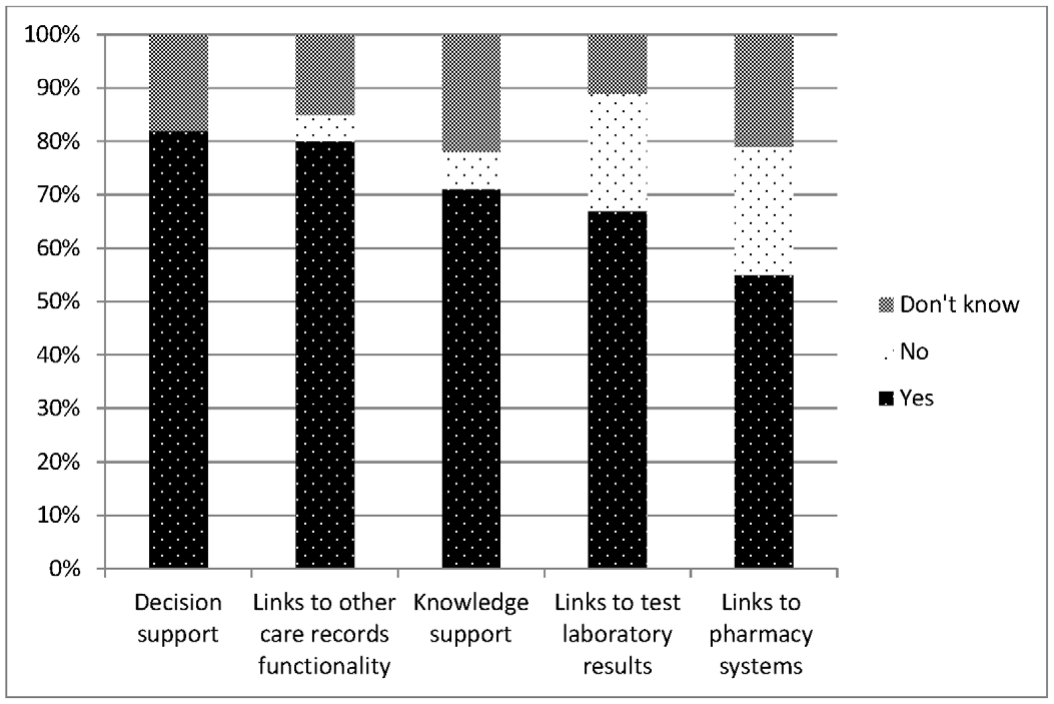
Graphical presentation of ePrescribing system functionality provided or expected to be provided.

In terms of system functionality provided or expected to be provided, 82% (n = 45) of Trusts indicated that they had or were planning to implement decision support functionality, 80% (n = 44) indicated computerised links to other patient care records functionality, 71% (n = 39) indicated knowledge support functionality, 67% (n = 37) computerised links to test laboratory results, and 55% (n = 30) computerised links to pharmacy systems (see Figure 2).

### Implementation Strategies and Early Lessons Learned

This section is divided into four themes and corresponding sub-themes emerging from the data. These will be discussed in turn and are summarised in Table 2 with illustrative quotes from participants. Although a range of issues were identified, many of these were common to different types of IT implementations in healthcare settings and are relatively well-known throughout the literature. [8], [13], [26]–[29] We will therefore focus on outlining those that are particularly relevant to ePrescribing systems implementations:

**Table 2:** Emerging themes and sub-themes from qualitative analysis of free text responses.

Themes	Sub-themes	Selected illustrative quotes and issues raised
**Strategic planning** **and testing of** **potential impact**	Implementation strategy	Often pilots and evaluations on selected wards before larger-scale roll-out (3–6 months prior), specialty areas often implement separately e.g. chemotherapy. Many implement as quickly as possible in one ward at a time (e.g. inpatients: between 1 and 4 wards every 2 to 4 weeks, most report planning to roll out in a year to implement in a medium to large hospital) to avoid running parallel paper and electronic systems as this is seen as risky. Throughout Trust (inpatient and outpatient) including full functionality, pilots and preparation: 3–5 years implementation average (from business case approval to full roll-out). The general strategy is to implement according to type of wards/specialty and roll-out across linked areas, many implement in specialty areas last. Most do inpatients first, then Accident and Emergency and outpatients, but some outpatients first as highest risk area. The tendency is to begin with simple functionality (e.g. to-take-out medication), roll this out and then add the more complex functionality (e.g. intravenous prescribing and medicines administration, other bespoke prescriptions e.g. sliding scale insulin) through system upgrades. Specialties first to implement: elderly care ward, oncology, outpatients, surgical ward.
	Piloting and testing	*“More testing/establishment of information and communication technology equipment”* *“Scope everything very thoroughly before making roll out decisions”*
	Realistic timeframes	*“Timescales difficult to achieve - set realistic ones”*
	Configuration and management ofdrug database	*“Lots of prep time to build drug file etc. is beneficial later on”*
	IT support and training	*“Understanding implementation support required”*
	An integrated strategy	*“Have a Trust strategy for all ePrescribing first”*
	IT infrastructure	*“Ensure IT infrastructure up to the job.”*
**Engagement and buy-in** **of different staff groups**	Different training needs/styles for different groups	
	Releasing staff from clinical dutiesto allow involvement indecision making	*“Too early except to say that "clinical buy in" has been difficult as time to attend demos is constrained by NHS commitments/activity i.e. medical and pharmacy staff cannot be released to attend lengthy seminars when the have clinics, theatre lists and ward rounds. Danger is final decision made by staff who may not be front end users.”*
	Lack of clinical input in systemschoice (including hardware) andbusiness case	*“Consensus is important amongst clinicians prior to beginning procurement”*
	Clinical champions	*“Medical and clinical leads to drive implementation and deployment.”*
	Lack of engagement from nurses	*“Work had to engage nurses who are reluctant to engage.”*
	Maintaining momentum in engagement	*“Using an extended team of clinicians and specialities was successful, but have now lost interest and need to re-energise”*
**Systems choice and** **desired functionality**	Need for standards	*“Need a clear criteria of functionality this is a must and which is evidence based.”*
	Interface design	*“Our focus is to use lean principles to drive quality. Hence less emphasis on barriers, restrictions, huge audit trail datasets. Instead focus on visibility, continuous information flow, measuring only sufficient data to deliver the message required! Hence design of interface is fundamental to safety and effectiveness.”*
	Support for mobile working	*“Most systems do not address mobile working and working in community which is important in mental health Trust and community areas.”*
	Realism - a perfect system doesnot exist	*“No system has all the answers - allow time for their developments”*
	Integration with existing systems	*“Good integration with other systems or integrated systems very important”*
	Desired functionality	(Better) integration with other local systems used across services (e.g. pathology, pharmacy, stock control) and primary care systems. Improved decision support functionality (e.g. intelligent alerting to prevent alert fatigue, including dose range checks, linked to clinical roles, support for infusions, adverse drug reaction and allergy checking, warfarin dosing algorithm, fluid therapy management, sliding scale insulin monitoring and adjustment). Good reporting and audit functionality. More intuitive user interface. System to be able to handle more complex medications and prescribing infusions, batch prescribing. Wireless and mobile working.
	System must meet individual organisational needs	*“Select a system that meets organisation needs (not necessarily part of a national programme)”*
	Systems choice is limited to companies who chose to bid	*“Limited by those who choose to those companies who choose to bid.”*
	System needs to be developed to a certain degree	*“Can’t wait for Lorenzo!”*
	Relationship with supplier	*“Specify the characteristics of the relationship with the supplier in the output based specifications.”*
**Top-level buy-in and management**	To adequately resource the project	*“Need resources to be able to utilise/roll out safely”*
	Cost-effectiveness and benefits realisation (evidence based)	*“There is no data out there demonstrating cost releasing savings - a key pillar for a successful business case”*
	Managing expectations whilst having a vision	*“Be optimistic - but realistic! Don’t be disheartened by pauses and challenges”*
	Benefits	Benefits hoped for: reduced prescribing/medication errors; greater efficiency in relation to medicines management processes (including discharge) and resulting time savings for staff and patients; improved patient safety; reduced cost (through better compliance with formularies and more streamlined processes); improved availability of data for audit and reporting; improved communication (across teams and over geographical distances) and more integrated medicines management process; reduction in medication administration errors and improved timeliness of administration; reduction in transcription errors and improved legibility; better audit trail; better accessibility of information; reduction in different charts used and paper; greater compliance with guidelines and pathways.
		Current benefits seen by those that have implemented or are in process of implementing:reduced medication prescribing errors; improved availability of data for audit; improved availability of information (e.g. no lost drug charts); improved alerts facilitating clinical decision making; improved adherence to guidelines; improved safety; mobile working; reduced medication administration errors and missed doses; improved communication between different departments; improved formulary support; improved legibility.
	Disbenefits	Workflow changes, increase in certain types of errors, time consuming for doctors and pharmacists.
	Flexibility in strategy	*“Flexibility versus structure”*
	Adequately sized project teams	Many mentioned that their management teams were too small and they found out at roll-out stage.
	Sharing lessons learned	*“We are in the process of building the case for ePrescribing so hope to learn from others and their experiences in this area.”*

- Strategic planning and testing of potential impact
- Engagement and buy-in of different staff groups
- Systems choice and desired functionality
- Top-level buy-in and management.

#### Strategic planning and testing of potential impact

Trusts that were planning to implement or in the process of procuring an ePrescribing system, expected to begin implementation in 2012 with an anticipated three to five year roll-out period. Between one and four wards were estimated to ‘go live’ (i.e. switch on the system) every two to four weeks. The underlying reasons for the nature of these roll-outs were attempts to minimise perceived safety risks surrounding the running of parallel paper and electronic systems in the same institution. Pilot implementations and extensive testing before larger-scale roll-out were viewed as important to identify and manage problems in a contained setting and predict the impact of new software and hardware on user workflows.

Most Trusts intended to begin with basic functionality (e.g. to-take-out medication), followed by roll-out across linked specialties, and implementation of more complex functionality (e.g. intravenous prescribing and other bespoke prescriptions such as sliding scale insulin). The most popular initial implementations were planned to be in elderly care and surgical wards, oncology, and outpatient settings. Underlying reasons for focusing on these areas included either that these were viewed as highly complex (i.e. it was assumed that if the system would work in these settings then it would be ‘easier’ to implement in the rest of the Trust), or as relatively ‘simple’ (i.e. they were used to identify problems before implementing in more complex settings). These may be perceived as contradictory arguments, which perhaps implies that participants recognised the possible challenges of implementation, but that there was no obvious case perceived across the sample for choosing one strategy over the other.

Despite these short implementation timelines, many participants reporting on their experiences of having implemented a system cautioned that the setting of realistic timeframes was important, illustrated by written statements such as *“don’t rush it, takes time to do it properly”* (Participant 7, Pharmacist) or *“don’t underestimate the time it will take”* (Participant 19, Pharmacist). A specific area mentioned as an example included configuration and management of the underlying drug database, where participants indicated that *“lots of prep time to build drug file etc. is beneficial later on”* (Participant 64, Pharmacist).

#### Engagement and buy-in of different staff groups

Due to the variety of professions using ePrescribing systems (i.e. clinicians including junior doctors, pharmacists, nurses), engaging different staff groups as early in the process as possible, preferably in relation to systems choice, was viewed as crucial, although often not realised:

*“Most systems lack clinical input and yet clinicians are expected to use systems and have little choice in system chosen.”* (Participant 18, Pharmacist)

An important constraint here was the difficulty of releasing staff from clinical duties to allow involvement in decision making:

*“…’clinical buy in’ has been difficult as time to attend demos is constrained by NHS commitments/activity i.e. medical and pharmacy staff cannot be released to attend lengthy seminars when they have clinics, theatre lists and ward rounds. Danger is final decision made by staff who may not be front end users.”* (Participant 3, Pharmacist)

Clinical champions were viewed as important to engage senior medical staff, but participants stated that some staff groups tend to be neglected, resulting in a lack of engagement. This was particularly true in relation to the most frequent users of the system including nurses and junior doctors.

#### Systems choice and desired functionality

The range of systems available, with different functionalities and of varying cost, resulted in a perceived need for criteria of functionality and technical standards to guide systems choice for Trusts:

*“In my journey so far it is very important to determine a clear set of criteria which all systems must comply with. This is huge problem.”* (Participant 18, Pharmacist)

Many participants also cautioned that an optimal system fulfilling requirements of all users and implementers was: 1) unlikely to exist; and 2) unlikely to be useful as “…*a system that does everything will possibly be a jack of all trades, master of none.”* (Participant 19, Pharmacist) Most existing systems were also seen as needing incremental development to suit the needs of hospitals, although some degree of maturity before procurement was viewed as necessary. As one respondent noted:

*“Don’t buy a product that is not developed yet (or even close to it)”* (Participant 60, Pharmacist)

Several desirable system functionalities were identified, including above all, the ability to interface with other local systems.

*“Pharmacy would like to see as much integration into other systems as possible - simple transformation of electronic drug charts to discharges and interfacing with pharmacy dispensing systems.”* (Participant 30, Pharmacist)

The desired functionality in relation to interfacing also included seamless integration with primary care systems: “Seamless integration with primary care prescribing, populating a single patient medication record that records what has been issued as well as prescribed.” (Participant 7, Pharmacist).

Many existing technologies lacked the functionalities and were viewed as too immature. This was also expressed in the hope for improved sophistication of decision support functionality, both in relation to handling a wider range of prescribing scenarios and in terms of preventing alert fatigue: *“More "less" decision support! i.e. more helpful but less frequent alerts”.* (Participant 61, Pharmacist).

Other desired functionalities included improved user interfaces, better support for wireless and mobile working (especially in community settings), and better reporting and audit functionality.

Despite the importance of systems choice, some participants mentioned that *“the relationship/culture of the supplier is even more important than the product”*. (Participant 20, Consultant Clinical Lead) This related to the on-going communication of user needs and the supplier’s response in terms of system development characterised by two-way communication channels between Trusts and suppliers.

*“Ensure at procurement to re-communication channels and ethos/stance on development direction”* (Participant 27, Manager)

The product specifications and the nature of the relationship with the supplier (including input in system development) set out at the procurement stage and determined within the contract were stated to be important to achieve this.

*“Software companies need things to be specified in the contract if you want them delivered. Tie up your contract tightly.”* (Participant 61, Pharmacist)

#### Top-level buy-in and management

Senior buy-in from hospital managers was described as being essential to ensure adequate project resourcing and safe implementation. The high costs of ePrescribing implementation and a lack of resources, including management teams that were often seen as being too small, were a concern to many participants.

*“Concerns - raising awareness of true resources to implement, manpower needed to optimise system use, interpret clinical/visit information potential of the system”* (Participant 9, Pharmacist)

*“Within a district general hospital the cost of implementation is relatively high. I feel it’s difficult for us to go to the board for funding. How can smaller hospitals keep up?”* (Participant 30, Pharmacist)

Once funded, managing expectations whilst keeping the aim in sight was viewed as essential. Participants stated that stakeholder expectations of ePrescribing systems were often higher than what was realistically achievable and that this needed to be communicated to all stakeholders involved, including senior management.

*“Manage expectations - people expect far more than EP* [ePrescribing] *can deliver”* (Participant 61, Pharmacist)

*“Expectations are high but reality is lower”* (Participant 65, Pharmacist)

*“Executive directors believe it will save money as no spare money for system - it must pay for itself. Need to tackle these fanciful ideas early on. Exec directors think it will do everything.”* (Participant 66, Pharmacist)

However, expectation management was seen to be complicated by the fact that data on cost-effectiveness and benefits realisation of systems was lacking. Such data were also often needed for developing a business case.

*“Data on benefits realisation/cost-effectiveness seems to be the main requirement for our finance team (as we put a business case together) rather than patient focus/patient safety.”* (Participant 41, Pharmacist)

*“There is no data out there demonstrating cost releasing savings - a key pillar for a successful business case”* (Participant 65, Pharmacist)

In terms of benefits, participants hoped for reduced errors, improved safety, greater efficiency and reduced cost. Benefits observed by those that had implemented ePrescribing included reduced errors, improved availability of data for audit, and fewer lost drug charts. However, Trusts that had already begun implementation also reported some adverse consequences, particularly in relation to workflows (which became more linear and less flexible), the introduction of other types of errors that were not present before the implementation, and the often time-consuming nature of system operation.

*“Change in type of errors occurring - not a lowering in error rate”* (Participant 61, Pharmacist)

*“Saving time? Too early to say” “Interestingly, drug rounds are taking longer so no efficiency seen as yet”* (Participant 25, Nurse Lead)

To address such unanticipated issues, persistence and flexibility in strategies on part of the management team was viewed as essential.

*“Persist, persist, persist! (Be willing to try different approaches)”* (Participant 36, Pharmacist)

## Discussion

### Summary of Main Findings

Our work indicates that, whilst few have begun implementation, many English Acute and Mental Health NHS Trusts are planning to implement ePrescribing systems with ambitious implementation timelines over the next three to five years. This is despite the apparent need, highlighted by those who have begun implementation, to allow sufficient time for piloting and testing, and to set more realistic timeframes. Although many Trusts are currently in the procurement stages, the majority were still unclear as to which system to choose. Amongst those that had a specific system in mind, the underlying reason was that the Trust had already implemented a system from the same supplier, which they hoped would facilitate integration with other systems and help to build on an established relationship with respective suppliers.

Many respondents highlighted the need for increased guidance in relation to implementation strategy, system choice and standards, as well as additional financial resources to fund local activities to support safe implementation. Whilst advocating realism in relation to system capabilities, desired functionalities included integration with existing local and primary care systems as well as more sophisticated decision support. Although some early benefits in relation to reduction of errors were realised in sites that had already implemented, the anticipated benefits relating to improved efficiency and cost-savings remain elusive.

### Strengths and Limitations of this Work

Our scoping study provides an overview of progress and plans to implement ePrescribing systems into English NHS hospitals as well as an insight into expectations and early lessons learned. The nature of the work, combining numerical, categorical and free-text entries, allowed an insight into progress and plans that may be extrapolated to a larger population, as well as providing an insight into underlying reasons and likely barriers that may inhibit progress in the future.

Participants came from 34% (55 of 168) of English Acute Trusts and represented a large range of geographical areas and demographics. Our results are likely to be broadly transferable to other Trusts, but inferences have to be made with caution as the majority of managers and pharmacists who attended the conference were interested in implementing ePrescribing systems. It is therefore likely that, as a result of drawing on a convenience sample, there may be lower interest in implementing ePrescribing systems in non-participating Trusts. Nevertheless, based on the number of Trusts represented, the numbers are substantial.

A further limitation relates to the design of our questionnaire, which was deliberately kept as short as possible to maximise the chances of it being successfully completed. This however meant we were not able to probe issues in detail and hence were unable to distinguish between current and future implementation–related activities.

### Considering our Findings in the Light of the Current Implementation Landscape

Two years after our first survey of planned implementations of ePrescribing systems in English hospitals [20], our current work has confirmed the increasing activity surrounding procurement and implementation with 55% of Trusts planning to implement and 20% currently implementing a system (total: 75%). These numbers are comparable to the 82% of Trusts who were either planning to implement or in the process of implementing an ePrescribing system in our initial survey, and an increase in ePrescribing system implementations internationally [32]. However, despite the remaining interest and activity, we can observe an increasing uncertainty in relation to systems choice reflected in the large number of different systems being actively considered. This may be due to the immaturity of existing systems, and lack of high-level strategic leadership demonstrated in our current work. Other countries have taken a different approach to implementation, which might address these issues to some extent. For example, the United States of America is promoting the principle of Meaningful Use, encouraging healthcare organisations to implement and adopt systems that fulfil national standards with the help of financial incentives [33], [34]. This approach is more likely to lead to competition amongst commercial providers, resulting in a “pool” of accredited systems that organisations can chose from whilst still having some element of autonomy in relation to functionality.

In the UK, it is now important that system suppliers and NHS stakeholders work together to develop existing systems further as these are currently relatively immature in English hospital settings [35]. Expectations are high particularly in relation to the inclusion of sophisticated decision support functionalities and interoperability with existing systems, as this is likely to ensure that technologies are effectively adopted and utilised to derive maximum benefits. However, unrealistic expectations surrounding the capabilities of systems may inadvertently result in disappointment and disillusioned stakeholders when faced with systems that are performing in ways other than desired [21], [36]. Such functionality already exists in international centres of excellence but requires extensive customisation [37], [38], which is relatively difficult to achieve with commercial systems as these need to satisfy a large range of needs. Building relationships with suppliers will be essential in order to achieve anticipated returns on investment and necessitates a careful balancing act between a system that suits individual settings and a solution that is commercially viable. Paradoxically, customisation is likely to result in trade-offs surrounding system maintenance and interoperability: the fact that a large range of different ePrescribing systems are available and planned to be implemented nationally could compromise exchange of information between systems [39]. This phenomenon is well-known in other organisational settings and existing work indicates that a certain degree of integration of local systems may nevertheless be possible [40].

Overall, although advocated by many in the past [41], [42], more realism is still needed in relation to implementing information technologies in health service organisations. ePrescribing system implementations are still in their infancy and despite some early benefits, systems necessarily have some drawbacks that need to be accommodated. It is of concern that implementing Trusts have reported the introduction of new errors, but this could be addressed by sharing lessons learned surrounding perceived short-comings and likely risks of different types of systems. This could then help Trusts that have yet to implement to plan for these in advance.

Planned timelines of Trusts yet to implement seem, in the light of limited resources and lack of demonstrated efficiency savings, to be perhaps rather ambitious. This is despite those with experience calling for more thorough planning and testing activity, which is by definition time-consuming. This is complicated by the fact that there is still limited knowledge surrounding the parallel running of paper and electronic systems both in practice and in the published literature [13], [21], [43]. However, our work has shown that parallel systems are a concern for many and an underlying reason for pushing implementations forward. Overall, the concern that paper and electronic systems used in parallel result in increased threats to patient safety may need to be secondary, when to considering the potential risks associated with rushed implementation timelines.

### Implications for Policy, Practice and Future Research

Our work has shown that there is a widespread recognition that ePrescribing systems have significant potential in improving the safety and quality of care and hence many Trusts are actively planning or pursuing implementation. However, with the demise of the NPfIT [21], [22], Trusts are now facing the choice amongst various systems with differing capabilities and costs, whilst lacking central guidance in relation to system standards and implementation strategies. The need for a central body facilitating the development of systems according to standards ensuring usability and interoperability is therefore apparent.

The present work is intended as a starting point of the journey to more widely implement ePrescribing in English NHS hospitals. We have recently commenced working on a national evaluation of ePrescribing systems in English hospitals, which aims to provide Trusts with an implementation toolkit and guidance in relation to systems choice [25]. In doing so, we plan to track developments over a period of five years and attempt to estimate the effectiveness and cost-effectiveness of various systems. However, our work is limited in time and resources and it is now important that longer-term strategies are developed to tackle the issue surrounding higher-level leadership.

In terms of research, our work suggests that there is an urgent need to examine the use of parallel electronic and paper systems as the perceived risks associated with this seem to drive ambitious implementation timelines. As a result, some effective ways to tackle parallel systems use may be devised, or it may be discovered that concerns are unfounded and timelines can be relaxed as a result.

### Conclusions

The majority of English Acute NHS Trusts in our sample are still in the process of planning to implement ePrescribing systems in the forthcoming years with fast-paced roll-outs, driven by high expectations surrounding improved safety and efficiency of care. However, the large range of existing systems, the lack of standards surrounding systems choice, restricted funding due to unclear return on investment, and the immaturity of existing systems has left many Trusts uncertain as to how to approach systems procurement.

Interoperability with existing systems and sophisticated decision support functionality are likely to increase the appeal of existing ePrescribing systems to Trusts, but these require a careful balancing act between customisation and setting of standards to allow exchange of information. In order to achieve this, a more integrated approach to sharing lessons and experiences is needed to help ensure that unanticipated issues can be planned for in advance and that ePrescribing systems fulfil the promise of safer medicines management in hospitals.

## Supporting Information

Figure S1 **Sample questionnaire - plans to and experiences of implementing ePrescribing systems in your hospital.**
(DOCX)
