# Correction: Investigating and Learning Lessons from Early Experiences of Implementing ePrescribing Systems into NHS Hospitals: A Questionnaire Study

**DOI:** 10.1371/annotation/1cec200a-6652-449f-91e9-8effb769de1e

**Published:** 2013-09-27

**Authors:** Kathrin Cresswell, Jamie Coleman, Ann Slee, Robin Williams, Aziz Sheikh, ePrescribing Programme Team

Text was erroneously omitted from Figure 1. The correct Figure 1 can be found here: http://www.plosone.org/corrections/pone.0053369.cn.g001.tif

