# Supplementary material for: Investigating and Learning Lessons from Early Experiences of Implementing ePrescribing Systems into NHS Hospitals: A Questionnaire Study
Source: PLoS One. 2013 Jan 15;8(1):e53369. doi: 10.1371/journal.pone.0053369 (PMC3546047; doi:10.1371/journal.pone.0053369)
Supplement: Figure S1 — Sample questionnaire - plans to and experiences of implementing ePrescribing systems in your hospital. (DOCX) [file pone.0053369.s001.docx]

**Figure S1: Sample questionnaire - plans to and experiences of implementing ePrescribing systems in your hospital**

**We’d be very grateful if you could please take a moment to complete this short confidential questionnaire, which is aimed at NHS staff. We are interested in understanding your hospitals plans/experiences of implementing ePrescribing systems and any key lessons that you have learned along the way.**

**We define ‘ePrescribing’ as: “*The utilisation of electronic systems to facilitate and enhance the communication of a prescription or medicine order, aiding the choice, administration and supply of a medicine through knowledge and decision support and providing a robust audit trail for the entire medicines use process.”***

**SECTION 1: ABOUT YOU AND YOUR HOSPITAL**

| **Which hospital do you work for?** ...............................................................................  **What is you role?** ............................................................................... | | | | | |
| --- | --- | --- | --- | --- | --- |
| **In relation to ePrescribing system(s) in your hospital… (Please tick one)** | | | | | |
| a | We have already an implemented ePrescribing system | 🞎 | c | We are planning to/procuring an ePrescribing system | 🞎 |
| b | We are currently implementing an ePrescribing system | 🞎 | d | We have no current plans to implement ePrescribing | 🞎 |

If you ticked a), b) or c) above please answer the questions in Sections 2 and 3. If you ticked d) please move straight on to Section 3 below.

**SECTION 2: ePRESCRIBING IN YOUR HOSPITAL**

| **When did/will your hospital start/complete implementation?** | | | | | | |
| --- | --- | --- | --- | --- | --- | --- |
| Start: ............................................year  Complete: ............................................year | | | | | | |
| **Which ePrescribing system has/will your hospital choose/chosen? (please tick)** | | | | | | |
| Ascribe | 🞎 | MedChart | | | 🞎 | |
|  |  | Don’t know | | | 🞎 | |
| Cerner | 🞎 | Built in-house? | | | 🞎  Name: | |
| HEPMA | 🞎 |  |  |  |  |  |
| JAC | 🞎 | Other *(please state)*? | | |  | |
| **Why did your hospital choose this system?** | | | | | | |
|  | | | | | | |
| **What functionality does/will your ePrescribing system provide?** | | | | | | |
| Knowledge support *(with immediate access to medicines information, e.g. BNF)?* | | | Yes  🞎 | No  🞎 | | I don’t know  🞎 |
| Decision support *(aiding the choice of medicines and other therapies, with alerts such as drug interactions)?* | | | 🞎 | 🞎 | | 🞎 |
| Computerised links to other elements of patients' individual care records *(e.g. Patient Administration System)?* | | | 🞎 | 🞎 | | 🞎 |
| Computerised links with laboratory results / test ordering? | | | 🞎 | 🞎 | | 🞎 |
| Computerised links to pharmacy systems? | | | 🞎 | 🞎 | | 🞎 |
| Other *(please state)*? | | | | | | |
| **Which specialty/ward areas was/will be the first to implement ePrescribing (e.g. type of early implementing wards)?** | | | | | | |
|  | | | | | | |
| **What was/is your subsequent roll-out plan and why?** | | | | | | |
|  | | | | | | |
| **What was/is your local implementation strategy?**  **Prompts:** **What is the scope of the implementation? What is the timeframe? How is functionality introduced?** | | | | | | |
|  | | | | | | |
| **What are the most significant changes you have seen/hope to see as a result of implementing ePrescribing in your hospital?**  **Prompts: Are, for example, professionals/the hospital more/less efficient?** | | | | | | |
|  | | | | | | |
| **In the journey towards implementing ePrescribing systems, what are your “top three” lessons learned to date?**  **Prompt: What would you do differently if you were to do it again?** | | | | | | |
| **1.**  **2.**  **3.** | | | | | | |

**SECTION 3: LOOKING AHEAD AND OTHER COMMENTS**

| **What functionality do you hope ePrescribing systems will provide in the future?** |
| --- |
|  |
| **Please use this space for any additional comments that you may have in relation to ePrescribing** |
|  |

**We are very grateful** **for your help. Please return the completed questionnaire to a member of the Conference team. For further details, please contact: Dr Kathrin Cresswell, eHealth Research Group, The University of Edinburgh (Kathrin.Beyer@ed.ac.uk**)
